# Objective physical activity level is associated with rectus femoris muscle echo‐intensity in patients with chronic obstructive pulmonary disease

**DOI:** 10.1111/crj.13528

**Published:** 2022-07-22

**Authors:** Kazuki Okura, Masahiro Iwakura, Atsuyoshi Kawagoshi, Keiyu Sugawara, Hitomi Takahashi, Takanobu Shioya

**Affiliations:** ^1^ Division of Rehabilitation Akita University Hospital Akita City Japan; ^2^ Department of Rehabilitation Akita City Hospital Akita City Japan; ^3^ Department of Physical Therapy, School of Health Sciences Fukushima Medical University Fukushima City Japan; ^4^ Geriatric Health Facility Nikoniko‐en Akita City Japan

**Keywords:** chronic obstructive pulmonary disease, echo‐intensity, physical activity, skeletal muscle, ultrasound imaging

## Abstract

**Introduction:**

Skeletal muscle dysfunction is one of the major extrapulmonary complications of chronic obstructive pulmonary disease (COPD). Some studies have reported a relationship between physical activity (PA) level and skeletal muscle quality assessed by echo‐intensity (EI) in healthy individuals but not in patients with COPD.

**Objectives:**

The aim of this study is to investigate the relationships between PA level and both skeletal muscle EI and skeletal muscle mass in patients with COPD.

**Methods:**

We employed a cross‐sectional design. Forty male outpatients with stable COPD were enrolled. Using B‐mode ultrasonography, we measured the rectus femoris muscle cross‐sectional area (RF‐CSA) and EI (RF‐EI). The RF‐CSA and RF‐EI were measured on frozen images using an electronic caliper and 8‐bit gray‐scale analysis, respectively. The objective PA level was determined by monitoring daily step counts and moderate‐to‐vigorous physical activity time (MVPA) with an activity monitor. A general regression model was used to assess the relationships between PA level and both RF‐CSA and RF‐EI. Age and body mass index (BMI) were adopted as confounding variables.

**Results:**

Twenty‐five outpatients with stable COPD (age, 70 ± 7 years old; forced expiratory volume in 1 s, 55.0 ± 24.9% of predicted values) were finally enrolled in the present study. Even after adjusting for age and BMI, the daily step counts and MVPA were significantly associated with RF‐EI, and knee extensor force was associated with RF‐CSA.

**Conclusion:**

The present study showed that PA level was associated with RF‐EI in patients with COPD. In addition, RF‐CSA was associated with knee extensor force. When assessing skeletal muscle using ultrasonography in patients with COPD, we should also assess EI.

## Graphical abstract

- We investigated the relationships between objective physical activity level and both skeletal muscle quality and mass in male patients with stable chronic obstructive pulmonary disease.
- We measured rectus femoris echo‐intensity as quality and cross‐sectional area as quantity using ultrasonography.
- Objective physical activity level was associated with rectus femoris echo‐intensity but not with those cross‐sectional area.

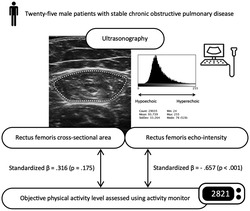

## 1 INTRODUCTION

Skeletal muscle dysfunction is one of the major extrapulmonary complications of chronic obstructive pulmonary disease (COPD), and lower muscle strength as well as pathophysiological changes have been reported.^1^, ^2^ Several studies focusing on skeletal muscle mass have reported that lower skeletal muscle mass is associated with mortality and readmission, as well as muscle strength.^3^, ^4^, ^5^ Recently, some studies have focused on skeletal muscle quality, which is often expressed as the content of noncontractile tissue such as intramuscular fat (or fatty infiltration) and is measured using computerized tomography (CT) or magnetic resonance imaging (MRI). Skeletal muscle quality can also be evaluated by echo‐intensity (EI), which is assessed using ultrasound imaging, a noninvasive, radiation‐free, and relatively quick procedure. Higher EI in the ultrasound image reflects greater noncontractile tissue such as intramuscular fat content and fibrosis tissue. EI has been reported to be associated with intramuscular fat content and fibrosis.^6^, ^7^ In patients with COPD, it has been reported that the EI was higher than that in healthy individuals and was associated with health‐related quality of life.^8^

The limitations of objective physical activity (PA) level have been reported in patients with COPD and have been associated with mortality.^9^ Therefore, interventions focused on PA promotion are an important component of pulmonary rehabilitation.^10^, ^11^ It has been reported that physical inactivity promotes skeletal muscle atrophy in patients with COPD,^12^ and therefore, assessment of skeletal muscle mass is important. However, no study has yet investigated whether a relationship exists between PA level and EI in patients with COPD. On the other hand, some studies have reported a relationship between PA level and EI in healthy individuals^13^, ^14^; thus; a similar result may exist in patients with COPD.

The purpose of the present study was to investigate the relationships between PA level and both skeletal muscle quality assessed by EI and skeletal muscle mass in patients with COPD.

## 2 MATERIALS AND METHODS

### 2.1 Study design and participants

A cross‐sectional design was used. All participants underwent measurements as follows: skeletal muscle ultrasound imaging, measurement of objective PA, measurement of physical functions, and measurement of pulmonary functions. According to the Global Initiative for Chronic Obstructive Lung Disease (GOLD) criteria, 40 male outpatients with mild‐to‐very severe COPD between October 2018 and March 2019 were enrolled.^15^ The exclusion criteria were as follows: an unstable condition with infection or exacerbation within at least the previous 3 months, use of any walking aid and/or wheelchair, neurological or musculoskeletal conditions that limit mobility inability, diagnosis of dementia or other mental disorders, and inability to operate the activity monitor.

### 2.2 Measurements

#### 2.2.1 Skeletal muscle ultrasound imaging

Ultrasound scanning was performed using a 14–5 MHz linear transducer with an ultrasound device (Noblus, Hitachi Aloka Medical, Co., Ltd., Japan) to acquire B‐mode ultrasound images. The ultrasound setups were kept constant during the study period: 9 MHz, 20‐dB gain, 75‐dB dynamic range, 6‐cm depth, and neutral time gain compensation. Only the depth focus was located on the rectus femoris muscle of each subject. The participants were in the supine position with the lower limbs relaxed completely. The transducer was placed on the superior aspect of the long axis of the thigh, two thirds of the distance from the anterior superior iliac spine to the superior patellar border.^16^ To be perpendicular to the targeted muscle, the angle of the transducer was fine‐tuned to show the best bone echo. Minimum pressure was maintained at the transducer, which was generously coated with a transmission gel to provide acoustic contact without compressing the skin or subcutaneous tissues. The ultrasound scanning was performed by a physical therapist with more than 5 years of scanning experience, who was not involved in the assessment of PA or other physical functions.

**FIGURE 1:**
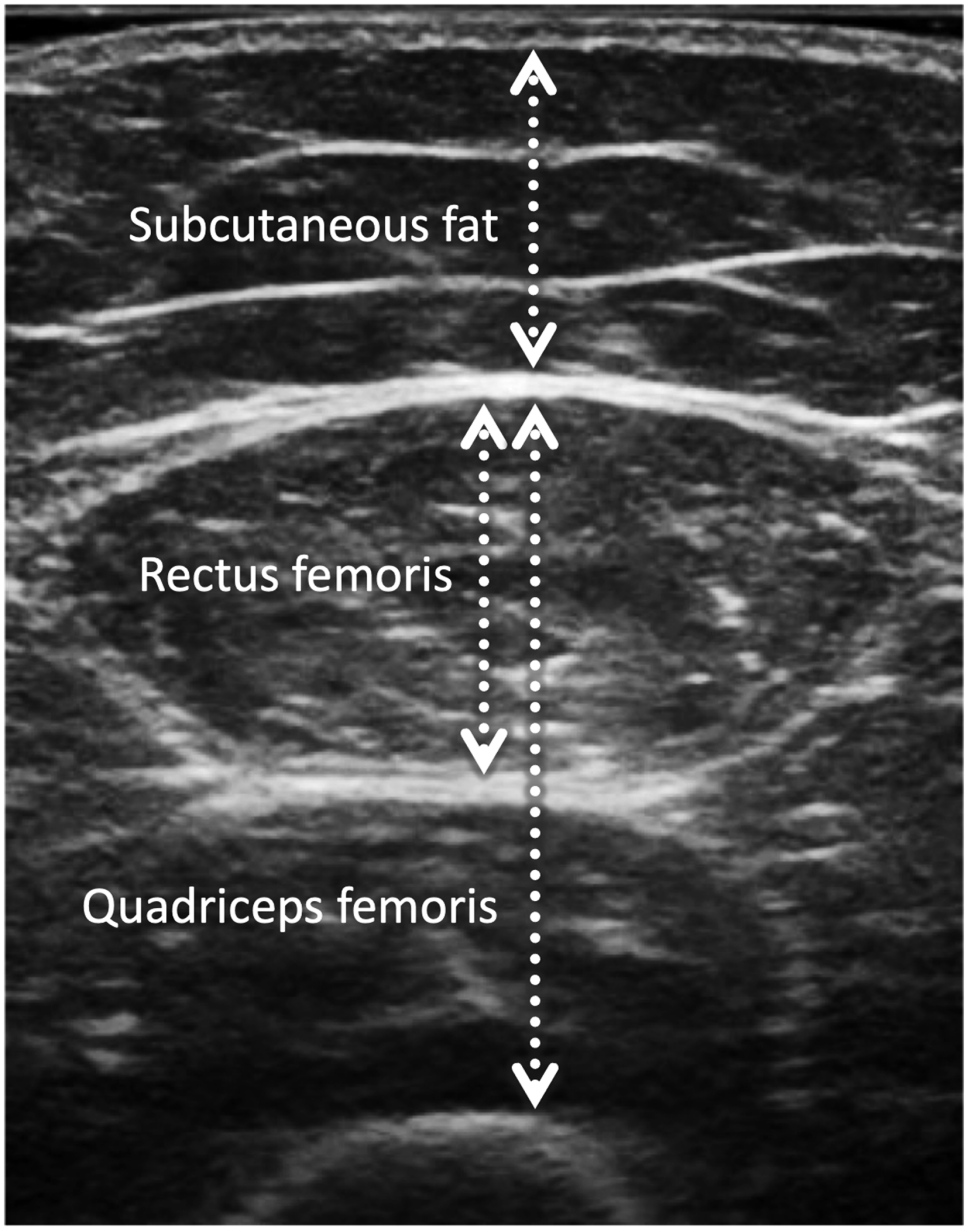
Ultrasonography measurement of subcutaneous fat thickness, rectus femoris thickness, and quadriceps muscle thickness

**FIGURE 2:**
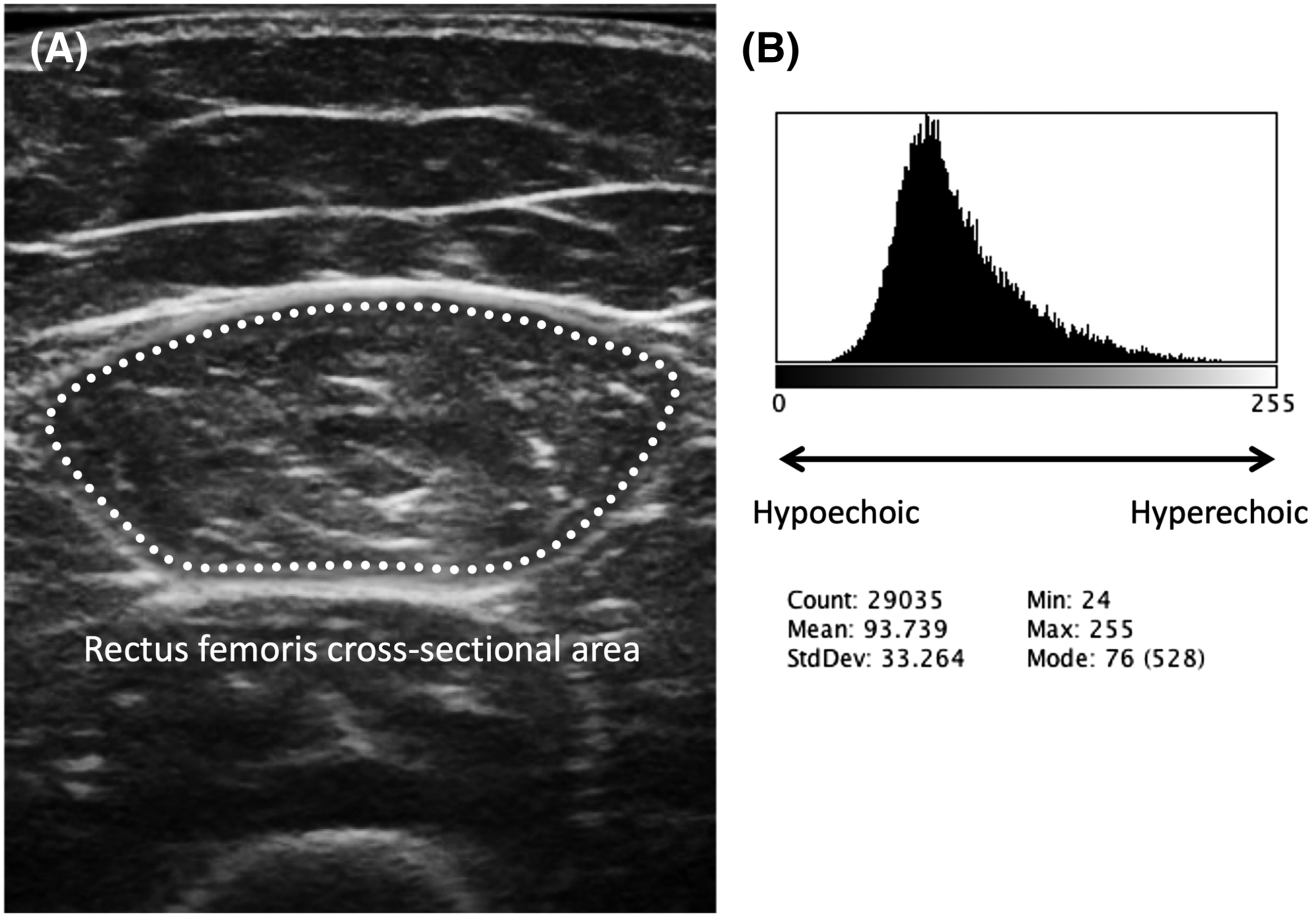
(A) Ultrasound measurement of the rectus femoris muscle cross‐sectional area (RF‐CSA). (B) Histogram of 8‐bit gray‐scale analysis of rectus femoris muscle echo‐intensity (RF‐EI). The region of interest was selected within the outline of the rectus femoris to include as much of the muscle as possible without any surrounding fascia.

The subcutaneous fat thickness of the anterior compartment of the thigh was defined as the distance between the dermis and superficial fascia of the rectus femoris muscle (Figure 1). The rectus femoris muscle thickness was measured as the vertical distance between the superficial and deep fascia (Figure 1). The quadriceps femoris muscle thickness was measured as the vertical distance from the superficial fascia of the rectus femoris to the underlying femur (Figure 1). These were measured on a frozen image using the electronic caliper function of an ultrasound device. The inner outline of the rectus femoris muscle was manually traced to calculate the rectus femoris muscle cross‐sectional area (RF‐CSA) on a frozen image (Figure 2A). Rectus femoris muscle echo‐intensity (RF‐EI) was measured using an 8‐bit gray‐scale analysis with ImageJ version 1.48 (National Institute of Health, USA).^17^ The region of interest was selected within the outline of the rectus femoris muscle, so as to include as much of the muscle as possible, without any surrounding fascia.^6^ The mean EI of the gray‐scale histogram was expressed in arbitrary units as a value between 0 (black) and 255 (white) (Figure 2B). The region of interest was determined three times for each image, and the median of the three consecutive measurements was used as the RF‐CSA and RF‐EI.

### 2.3 PA

Objective PA level was measured using an accelerometer‐based activity monitor (Lifecorder GS4, Suzuken, Co., Ltd., Japan), the validity and reliability of which have been demonstrated previously.^18^ The participants wore the monitor on the belt or waistband of their clothing for at least nine consecutive days. The device was worn throughout the day and was only taken off during bathing and sleeping at night. At least 7 days' worth of data on the participants' daily activities were obtained. Days where the device was worn for at least 10 h were considered valid. The participants' PA levels were determined by measuring the average daily step count and the time spent in moderate‐to‐vigorous physical activity (MVPA) per day. MVPA was defined as exercising at an intensity of at least three metabolic equivalents.

### 2.4 Physical functions

Handgrip strength was measured using a digital dynamometer (Grip‐D, Takei Scientific Instruments Co., Ltd., Japan). Two measurements of the dominant hand's maximum strength were taken using the dynamometer, and the highest value was recorded as the handgrip strength.

Maximal knee extensor force was measured using a stationary digital dynamometer (Hydromusculator GT‐160, OG Giken, Co., Japan). Measurements were taken with the patient in a sitting position, with their hips and knees at 90°. Knee extensor force was defined as the highest mean force that could be sustained for longer than 1 s. All measurements were repeated at least twice. A rest period of 60 s was provided between each contraction to allow the participants to recover from each effort. The highest force was recorded as the maximal knee extensor force.

A 10‐m walking test was performed to measure gait speed. The participants were instructed to walk at a comfortable pace. The test was performed twice for each subject, with a 10‐s break between the trials. The faster speed between the two trials was used as the gait speed.

A 6‐min walking test (6MWT) was performed in accordance with the European Respiratory Society (ERS) and American Thoracic Society (ATS) technical standards.^19^ The 6MWD was recorded as the patient's exercise capacity. The participants practiced for 1 min before performing the test once.

### 2.5 Pulmonary function and demographic data

Pulmonary function was assessed as forced vital capacity (FVC), forced expiratory volume in 1 s (FEV_1_), and FEV_1_/FVC measured using a spirometer (CHESTGRAPH HI‐701, Chest MI, Inc., Japan) by well‐trained physical therapists following the ATS guidelines.^20^ The FVC and FEV_1_ are expressed as percentages of the predicted values.^21^ Airflow obstruction was classified using the GOLD stage.^15^

The participants' age, height, weight, body mass index (BMI), and percentages of the ideal body weight (%IBW) were retrieved from clinical records. Modified Medical Research Council (mMRC) dyspnea scores were obtained as markers of the degree of dyspnea.

### 2.6 Statistical analysis

The statistical analysis was performed using R version 3.6.1 (The R Foundation for Statistical Computing) and RStudio version 1.2.5042 (RStudio, Inc., USA). *P*‐values less than 0.05 were considered statistically significant. The assumption of normality was assessed graphically and using the Shapiro–Wilk test.

We first conducted univariate analyses based on the general linear regression model using PA level (daily step counts and MVPA) and each of the potentially associated factors of RF‐CSA and/or RF‐EI (age, BMI, FVC, FEV_1_, knee extensor force, gait speed, and 6MWD) as independent variables and RF‐CSA and RF‐EI as dependent variables. Next, age and BMI were adopted as confounding variables and added to a multivariate analysis based on the general linear regression model.

## 3 RESULTS

Fifteen out of the initial 40 patients were excluded for the following reasons: two had recently exacerbated conditions; four had respiratory disease, in addition to COPD, that affected their PA (i.e., neuromuscular disease); three were unable to walk independently; one was unable to operate the activity monitor because of moderate dementia; and five declined to participate. Therefore, a total of 25 patients with COPD were included in the current study.

A summary of the participants is shown in Table 1. Sixteen percent (*n =* 4) of the participants were <65 years old, 28% (*n =* 7) were ≥75 years old, 24% (*n =* 6) had a low body weight (%IBW < 90%), 16% (*n =* 4) had weak handgrip strength (<27 kgf), 16% (*n =* 4) had a slow gait speed (<1.0 m/s), 8% (*n =* 2) had a low exercise capacity (6MWD < 350 m), 64% (*n =* 16) were physically inactive (daily step count <5000 steps/day), and 72% (*n =* 18) did not meet the WHO recommendations (MVPA <150 min/week). Skeletal muscle ultrasound imaging results are shown in Table 2.

**TABLE 1:** Characteristics of participants

Variable	Unit	Value
Age	years	70 (7)
Height	cm	166.6 (6.1)
Weight	kg	61.8 (7.8)
BMI	kg/m^2^	22.3 (2.9)
%IBW	%	101.3 (13.2)
FVC	L	3.31 (0.84)
	%predicted	96.2 (20.1)
FEV_1_	L	1.45 (0.69)
	%predicted	54.9 (24.9)
FEV1/FVC	%	42.8 (14.4)
GOLD stage		
I	*n*	4 (16)
II	*n*	9 (36)
III	*n*	8 (32)
IV	*n*	4 (16)
mMRC dyspnea scale		
0	*n*	3 (12)
1	*n*	12 (48)
2	*n*	6 (24)
3	*n*	4 (16)
4	*n*	0 (0)
Handgrip force	kgf	34.7 (6.9)
Knee extension force	kgf	44.2 (11.6)
	kgf/weight (kg)	0.71 (0.17)
Gait speed	m/s	1.13 (0.15)
6MWD	m	474.9 (99.2)
Daily step counts	steps/day	4501 (3354, 8474)
MVPA	min/day	12.2 (7.5, 26.4)

*Note*: *n =* 25. Values are expressed mean (SD), median (25th, 75th), or *n* (%).

Abbreviations: BMI, body mass index; FEV_1_, forced expiratory volume in 1 s; FVC, forced vital capacity; GOLD, global initiative for chronic obstructive lung disease; IBW, ideal body weight; mMRC; modified medical research council; 6MWD, 6‐min walking distance; MVPA, moderate‐to‐vigorous physical activity time.

**TABLE 2:** Measurements of ultrasound imaging

Variable	Unit	Value
Thickness		
Subcutaneous fat	mm	4.9 (2.1)
Rectus femoris	mm	12.6 (2.9)
Quadriceps femoris	mm	24.9 (4.8)
RF‐CSA	cm^2^	3.81 (1.19)
RF‐EI	Arbitrary unit	102.5 (10.1)

*Note: n =* 25. Values are expressed mean (SD).

Abbreviations: RF‐CSA, rectus femoris cross‐sectional area; RF‐EI, rectus femoris echo‐intensity.

The general regression model showed that age, knee extensor force, 6MWD, and daily step counts were associated with RF‐CSA, and BMI, gait speed, daily step counts and MVPA were associated with RF‐EI (Table 3). After adjusting for age and BMI, only knee extensor force was associated with RF‐CSA, and daily step counts and MVPA were associated with RF‐EI (Table 3).

**TABLE 3:** Results of general linear regression model

	Variables	Unadjusted							Adjusted^a^						
		95%CI							95%CI						
		B	SE	LCL	UCL	*β*	*t*‐value	*p*‐value	B	SE	LCL	UCL	*β*	*t*‐value	*p*‐value
RF‐CSA	Age	−0.084	0.032	−0.150	−0.018	−0.483	−2.648	0.014							
	BMI	0.029	0.086	−0.149	0.206	0.069	0.333	0.742							
	FVC	0.006	0.012	−0.020	0.031	0.100	0.483	0.634	0.006	0.012	−0.019	0.031	0.104	0.518	0.610
	FEV_1_	0.008	0.010	−0.012	0.029	0.177	0.860	0.399	0.009	0.009	−0.011	0.028	0.178	0.928	0.364
	Knee extensor force	4.761	1.137	2.409	7.113	0.658	4.187	0.000	4.001	1.248	1.407	6.596	0.553	3.207	0.004
	Gait speed	2.340	1.646	−1.065	5.746	0.284	1.422	0.169	1.093	1.663	−2.365	4.551	0.133	0.657	0.518
	6MWD	0.006	0.002	0.002	0.010	0.499	2.761	0.011	0.004	0.002	−0.001	0.009	0.372	1.979	0.061
	Daily step counts	0.000	0.000	0.000	0.000	0.483	2.645	0.014	0.001	0.001	−0.001	0.001	0.316	1.405	0.175
	MVPA	0.028	0.016	−0.004	0.061	0.351	1.800	0.085	0.013	0.017	−0.022	0.049	0.165	0.785	0.441
RF‐EI	Age	0.480	0.292	−0.124	1.083	0.324	1.644	0.114							
	BMI	1.631	0.646	0.295	2.967	0.466	2.525	0.019							
	FVC	0.037	0.105	−0.180	0.254	0.074	0.354	0.727	0.143	0.091	−0.046	0.332	0.283	1.576	0.130
	FEV_1_	−0.002	0.085	−0.177	0.174	−0.004	−0.018	0.986	0.049	0.074	−0.104	0.203	0.121	0.666	0.513
	Knee extensor force	−22.790	11.907	−47.421	1.841	−0.371	−1.914	0.068	−17.126	11.533	−41.110	6.859	−0.279	−1.485	0.152
	Gait speed	−28.554	13.311	−56.091	−1.018	−0.408	−2.145	0.043	−21.820	12.497	−47.810	4.169	−0.312	−1.746	0.095
	6MWD	−0.024	0.021	−0.067	0.019	−0.233	−1.149	0.262	−0.014	0.019	−0.054	0.026	−0.136	−0.717	0.481
	Daily step counts	−0.002	0.001	−0.004	−0.001	−0.660	−4.214	0.000	−0.002	0.001	−0.004	−0.001	−0.657	−3.911	0.001
	MVPA	−0.391	0.117	−0.633	−0.149	−0.571	−3.338	0.003	−0.403	0.105	−0.622	−0.185	−0.590	−3.836	0.001

*Note*: *n =* 25.

Abbreviations: *β*, standardized partial regression coefficient; B, partial regression coefficient; BMI, body mass index; 95%CI, 95% confidence interval; FEV_1_, forced expiratory volume in 1 s; FVC, forced vital capacity; LCL, lower limit of 95% confidence interval; RF‐CSA, rectus femoris cross‐sectional area; RF‐EI, rectus femoris echo‐intensity; SE, standard error; 6MWD, 6‐min walking distance; MVPA, moderate‐to‐vigorous physical activity time; UCL, upper limit of 95% confidence interval.

^a^ Age and BMI were adopted to model as confounding variables.

## 4 DISCUSSION

The present study investigated the relationships between PA level and both skeletal muscle quality measured by EI and skeletal muscle mass in patients with COPD. After adjusting for age and BMI, daily step counts and MVPA were found to be associated with RF‐EI. On the other hand, the association between PA levels and the RF‐CSA was weak and not statistically significant; only knee extensor strength was associated with the RF‐CSA.

The results of the present study indicate that RF‐EI was associated with daily step counts and MVPA in patients with COPD. Several studies have assessed muscle quality using image‐based methods and found that patients with COPD had more intramuscular fatty infiltration than healthy subjects.^22^, ^23^ However, only a few studies have examined the relationship between skeletal muscle quality and PA in patients with COPD. In healthy older adults, the association between EI as muscle quality and PA level has been reported cross‐sectionally and longitudinally.^13^, ^14^ Maddocks et al. conducted one of the few studies examining the association between muscle quality and PA level in patients with COPD and reported that fatty infiltration and CT attenuation were associated with PA level.^23^ A similar association was observed in the present study between PA level and skeletal muscle quality, using EI. Ultrasound imaging is a noninvasive, radiation‐free, relatively quick procedure, and is therefore excellent for repeated measurements regardless of location and situation, such as in the acute care and home care settings. Thus, it is significant that the results are similar to those of Maddocks et al.'s study. Both muscle quality and PA levels have been reported to decline from an early phase of COPD.^8^, ^24^ Increased intramuscular fat content in the quadriceps muscles has been reported to be associated with abnormal anaerobic metabolism in patients with COPD.^22^ In addition, increased intramuscular fat content has been reported to be associated with insulin resistance, which in turn has been reported to be associated with decreased knee extensor strength in patients with COPD.^25^ In healthy individuals, it has been reported that interventions focusing on PA level promotion inhibit the increase of intramuscular fat content.^26^ Thus, interventions focusing on promoting PA level, especially from the early phase of COPD, may contribute to maintaining muscle quality and physical function in patients with COPD, which is a subject for further study.

The relationship between skeletal muscle mass and strength has been reported in several studies on COPD, and the findings of the present study support this relationship.^12^, ^16^, ^27^ It has been reported that both skeletal muscle mass and strength are associated with long‐term prognosis and are important outcomes for patients with COPD.^3^, ^4^, ^5^ On the other hand, there was no strong association between RF‐CSA and daily step counts or MVPA in the present study. Some previous studies have reported an association between RF‐CSA and PA measured by ultrasound imaging; Shrikrishna et al. found a weak correlation between CSA and PA level in patients with COPD.^12^ However, they validated the results by separating GOLD stage I from GOLD stage II to stage IV and reported an association between RF‐CSA and PA level in GOLD stage I, but the same association was weak in GOLD stage II to stage IV, suggesting that the relationship can vary depending on COPD stage.^12^ Therefore, the present study was characterized by a small number of mild and very severe cases, which may have affected the association between RF‐CSA and PA level.

There are several limitations to the present study. First, our participants were voluntarily recruited from a single‐center cohort with several biases in the characteristics. In addition, the sample size was small, which made it difficult to determine the certainty of weak correlations, and multiple regression analysis was not feasible. This limitation may be one of the factors that prevented us from detecting the association between PA level and muscle mass and that between muscle strength and quality, as reported in previous studies.^12^, ^23^ However, we can refer to the possibility that these associations are weaker than the association between muscle strength and muscle mass and that between PA level and muscle quality found in the present study. Second, all participants were male. Mizuno et al. reported that, in healthy subjects, longitudinal changes in quadriceps muscles mass and CT attenuation were different between males and females.^28^ Furthermore, female COPD patients have been reported to have less muscle mass, higher EI, and less PA.^8^ Therefore, it is unclear whether similar results can be obtained in female subjects. Third, most of the participants had moderate‐to‐severe airflow obstruction. Shrikrishna et al. reported a difference in the strength of the association between RF‐CSA and PA between GOLD stage I and GOLD stage II to stage IV in patients with COPD.^12^ Therefore, there may be similar differences in the association between skeletal muscle quality and PA level depending on disease stage. In a study by Maddocks et al. that examined the relationship between muscle quality assessed by CT and PA, there were fewer participants in the early stage of COPD.^23^ Lastly, the participants in the present study had higher physical function (i.e., muscle strength and exercise capacity) on average than those in previous studies.^16^, ^23^ While previous studies have reported an association between muscle quality and physical function,^16^, ^23^ no association was found between RF‐EI and knee extensor force or 6MWD in the present study. The reason for this could be due to baseline differences. The subjects in a study by Ye et al., who measured EI in a similar method to that used in the present study, also had high exercise capacity (6MWD) and reported no association between EI and exercise capacity.^8^

We were unable to establish a longitudinal relationship between EI and PA level due to the cross‐sectional design of the study. Future longitudinal observation and intervention studies should be conducted to investigate the relationships between changing muscle quality (i.e., EI) and other clinical outcomes. In older inpatients of a subacute and convalescent rehabilitation hospital, the intramuscular adipose tissue contents in the quadriceps muscles found using CT were negatively associated with recovery of basic activity of daily living.^29^ Therefore, more intensive exercise training may be necessary for COPD patients who have poor skeletal muscle quality.

In conclusion, the results of the present study showed that PA level is associated with RF‐EI, but the association between PA levels and the RF‐CSA is weak and not statistically significant in patients with COPD. However, only knee extensor force was associated with the rectus femoris muscle cross‐sectional area. When assessing skeletal muscle using ultrasonography in patients with COPD, we should focus not only on muscle mass but also on EI.

## CONFLICT OF INTEREST

The authors declare that they have no competing financial interests or personal relationships that could have influenced the work reported in the present paper.

## ETHICS STATEMENT

This study was approved by the medical ethics committee of Akita City Hospital, 2017 (approval No.6) and was carried out in conformity with the Declaration of Helsinki. The objective and content of the study were explained orally to the participants, as well as in written documents. Written consent was obtained after the participants were informed that they could decide whether to participate based on their own free will and that their privacy would be reasonably protected.

## AUTHOR CONTRIBUTIONS

Designed study: Kazuki Okura, Keiyu Sugawara, Hitomi Takahashi, and Takanobu Shioya. Collected data: Kazuki Okura, Masahiro Iwakura, and Atsuyoshi Kawagoshi. Analyzed data: Kazuki Okura and Masahiro Iwakura. Drafted manuscript: Kazuki Okura. Reviewed manuscript: Kazuki Okura, Masahiro Iwakura, Atsuyoshi Kawagoshi, Keiyu Sugawara, Hitomi Takahashi, and Takanobu Shioya.

## Data Availability

The data used in the present study are available from the corresponding author upon reasonable request.
